# Supplementary figures and images for: Toll-like receptor 4-mediated inflammation triggered by extracellular IFI16 is enhanced by lipopolysaccharide binding
Source: PLoS Pathog. 2020 Sep 9;16(9):e1008811. doi: 10.1371/journal.ppat.1008811 (PMC7505474; doi:10.1371/journal.ppat.1008811)

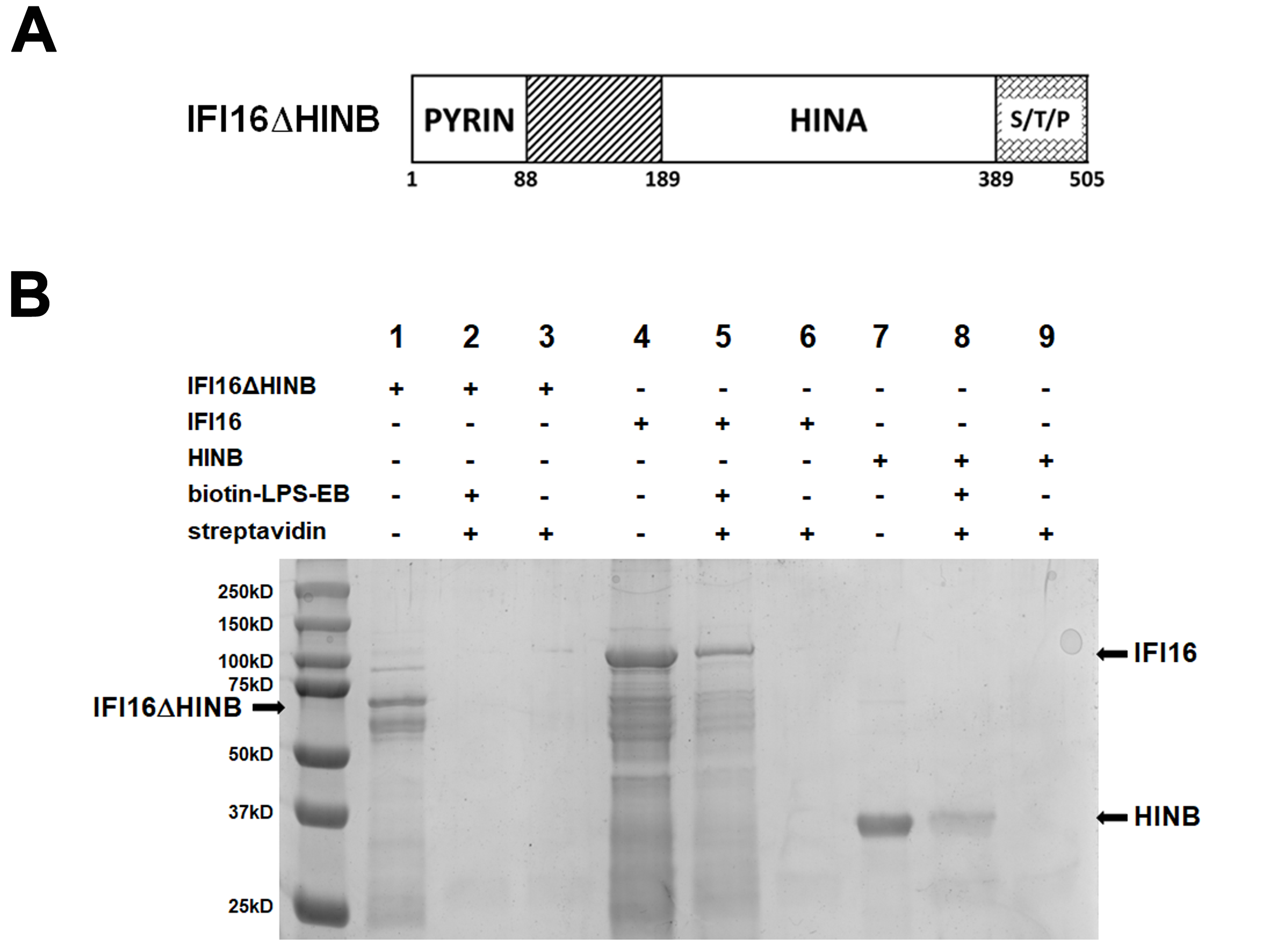

Supplement: S1 Fig — (A) Domain organization of the IFI16ΔHINB protein. The numbers represent the amino acid positions based on NCBI Reference Sequence NP_005522. From the N- to the C-terminal (left to right), IFI16ΔHINB comprises a pyrin domain, and only one hematopoietic interferon-inducible nuclear protein with 200-amino-acid repeats (HINA) domain. S/T/P = serine/threonine/proline-rich repeats. (B) Coomassie brilliant blue staining of pull-down assays performed with 3 μg of recombinant IFI16ΔHINB, full-length IFI16, or HINB domain in the presence or absence of biotin-labeled LPS from E. coli O111:B4 (biotin-LPS-EB). (TIF) [file ppat.1008811.s001.tif]

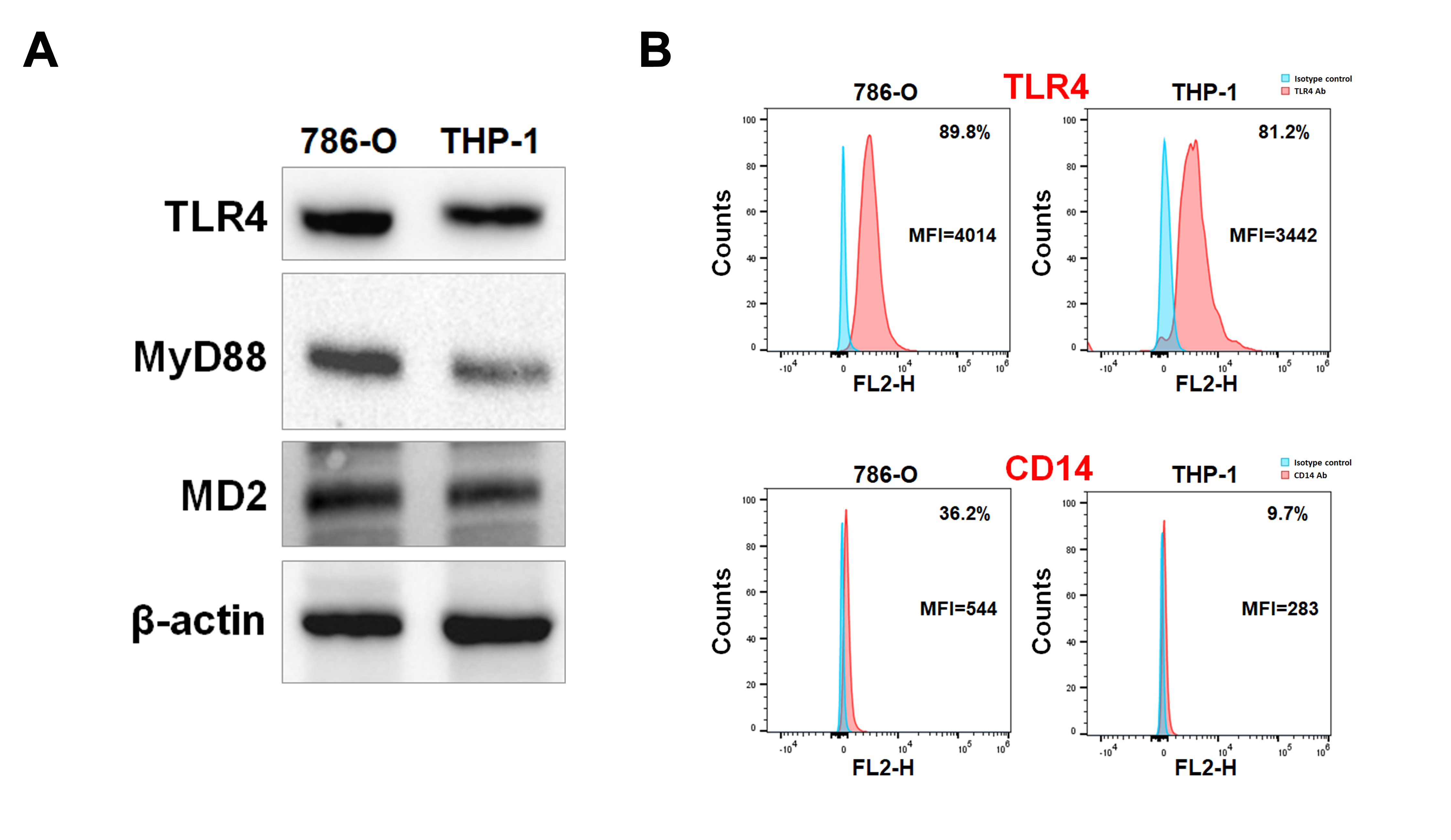

Supplement: S2 Fig — (A) Western blot analysis of TLR4, MD2 and MyD88 in whole-cell lysates of 786-O cells or THP-1 cells. Immunoblot with anti-β-actin antibody was used as loading control. (B) Cell surface expression of TLR4 and CD14 in 786-O and THP-1 cells (left and right panels, respectively), detected by flow cytometry using specific antibodies. Blue histograms represent background fluorescence; red histograms denote TLR4 (upper panels) or CD14 (lower panels) staining. The y-axis represents the number of cells, while the x-axis represents the level of fluorescence (FL-2) in a logarithmic scale. Images are representative of two independent experiments with similar results. The percentage of stained cells is reported in each panel. MFI = mean fluorescence intensity. (TIF) [file ppat.1008811.s002.tif]

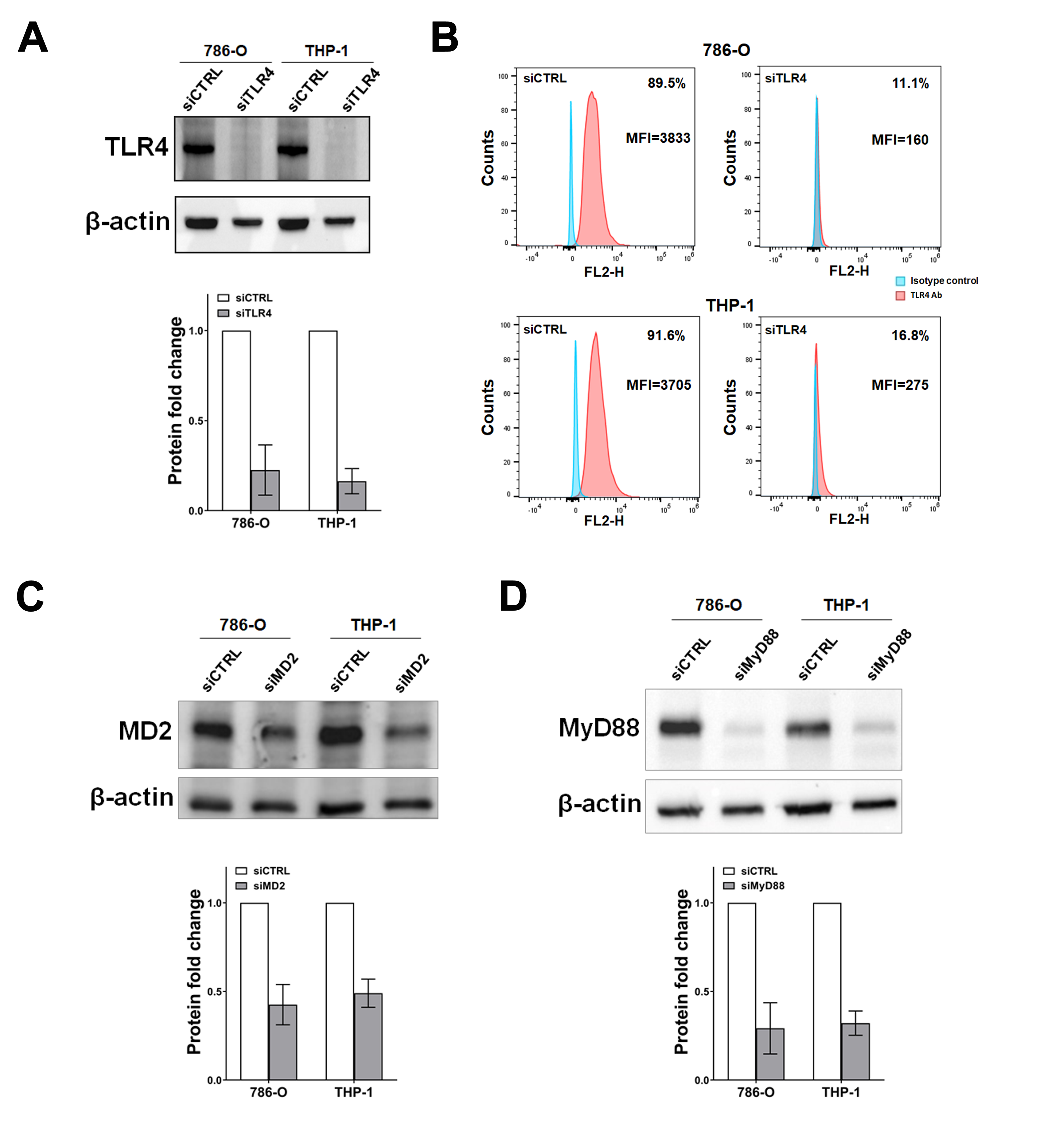

Supplement: S3 Fig — (A) Upper panel: Western blot analysis of TLR4 and β-actin in whole-cell lysates of 786-O cells or THP-1 cells transfected with non-targeting siRNA control (siCTRL) or siRNA TLR4 (siTLR4) at 48 h after transfection. Lower panel: densitometric analysis showing the fold change expression of the indicated proteins expressed as the mean from three independent experiments. Error bars indicate SD. (B) Cell surface expression of TLR4 in 786-O and THP-1 cells (upper and lower panels, respectively), detected by flow cytometry using specific antibodies at 48 h after transfection with siCTRL or siTLR4. Blue histograms represent background fluorescence; red histograms denote TLR4 staining. The y-axis represents the number of cells, while the x-axis represents the level of fluorescence (FL-2) in a logarithmic scale. Images are representative of two independent experiments with similar results. The percentage of stained cells is reported in each panel. MFI = mean fluorescence intensity. (C, D) Upper panels: Western blot analysis of MD2 (C) and MyD88 (D) in whole-cell lysates from 786-O cells or THP-1 cells transfected with non-targeting siRNA control (siCTRL) or specific siRNAs—siMD2 and siMyD88, respectively—at 48 h after transfection. Lower panels: densitometric analysis showing the fold change expression of the indicated proteins expressed as the mean from three independent experiments. Error bars indicate SD. (TIF) [file ppat.1008811.s003.tif]

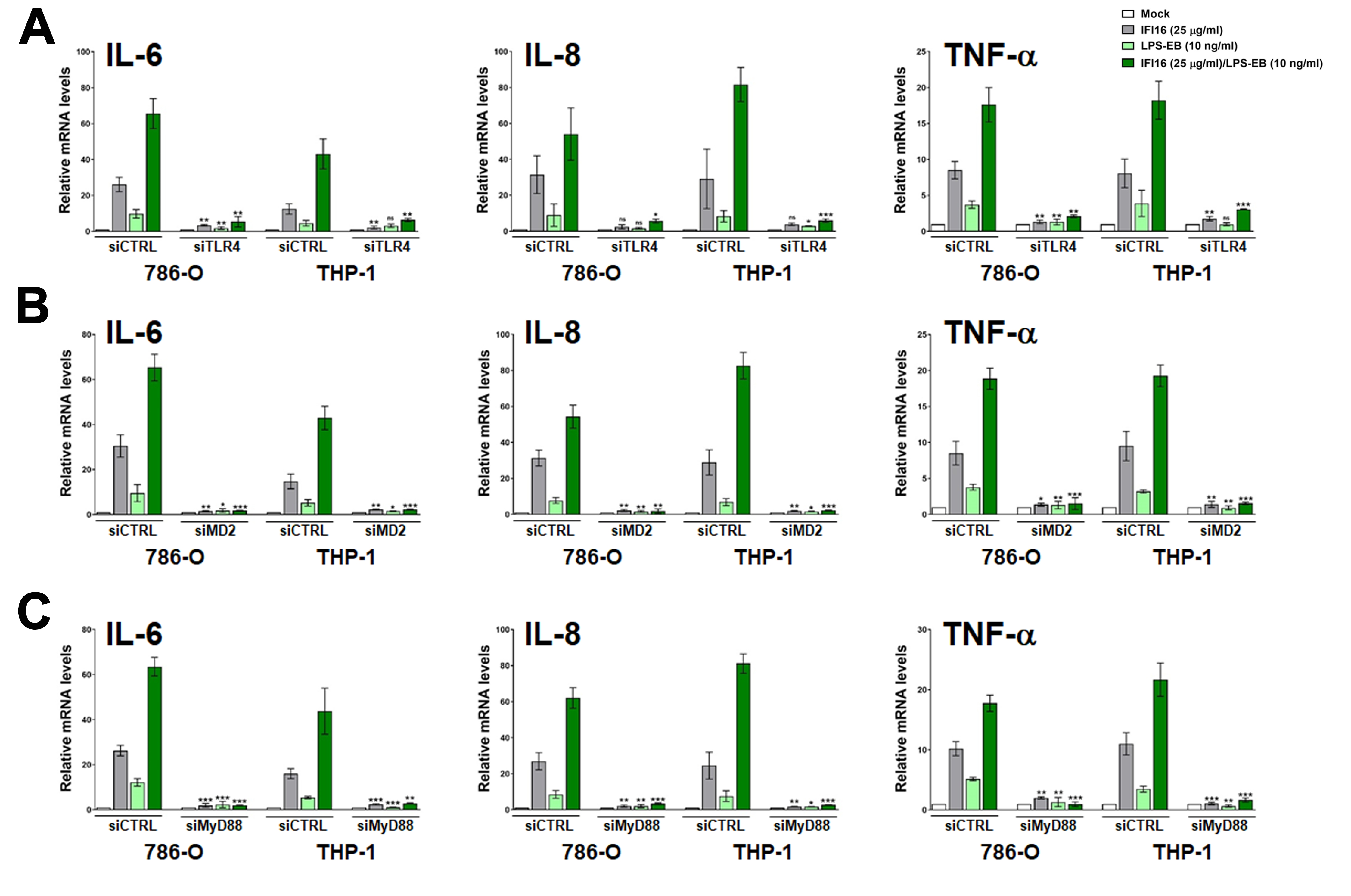

Supplement: S4 Fig — (A-C) qRT-PCR analysis of IL-6, IL-8 and TNF-α mRNA expression levels in 786-O or THP-1 cells transfected for 48 h with scramble control (siCTRL), or siRNAs against TLR4 (siTLR4) (A), MD2 (siMD2) (B) or MyD88 (siMyD88) (C). Cells were then stimulated for 24 h with IFI16 (25 μg/ml), LPS from E. coli O111:B4 (LPS-EB, 10 ng/ml) or IFI16/LPS-EB complex (preincubated O/N at 4°C), or left untreated (mock). Values were normalized to GAPDH mRNA and plotted as fold induction over mock-treated cells. qRT-PCR data are expressed as mean values of biological triplicates. Error bars indicate SD (*P < 0.05, **P < 0.01, ***P < 0.001, ns: not significant; unpaired Student’s t-test for comparison of silenced cells vs. their relative control counterpart). (TIF) [file ppat.1008811.s004.tif]
