# Supplementary material for: Toll-like receptor 4-mediated inflammation triggered by extracellular IFI16 is enhanced by lipopolysaccharide binding
Source: PLoS Pathog. 2020 Sep 9;16(9):e1008811. doi: 10.1371/journal.ppat.1008811 (PMC7505474; doi:10.1371/journal.ppat.1008811)
Supplement: Attachment — Submitted filename: Iannucci et al_Response to Reviewers.docx [file ppat.1008811.s005.docx]

**Part I Summary**

***Reviewer #1****: In the present study, the authors followed up on their previous observation that IFI16 is detectable in sera from immune-activated individuals and that recombinant IFI16 boosts inflammatory responses in cell culture. Here, they examined the underlying mechanism of this effect of IFI16. They present evidence that recombinant IFI16 binds to LPS on the surface of Gram- bacteria, regardless of their potency in activating TLR4 signaling. In addition, they found that the lipid A component of LPS and the HIN-B domain of IFI16 play important roles in this interaction. Finally, the authors further show that IFI16 complexed with the LPS displays a slightly higher ability than IFI16 alone to trigger immune activation via the TRAP/MyD88/NF-κB pathway. Finally, Iannucci and colleagues report that IFI16 directly binds to TLR4 and that the binding affinity of the IFI16/LPS complex is higher compared to the LPS alone. In conclusion, they propose that IFI16 may be a novel DAMP that potentiates the action of TLR4 by promoting its signaling by binding to subtoxic doses of LPS.*

*The study is competently performed and the results potentially interesting. The major limitation of the present (and previous) study is that all results were obtained using bacterial expressed tagged IFI16. Usually, IFI16 shows a nuclear localization and it is difficult to assess how the concentrations of circulating IFI16 achieved in vivo compared to those used in the present study. In addition, the possibility that endogenous and bacterial expressed recombinant IFI16 show structural and functional differences cannot be excluded.*

***Reviewer #2****: This study shows that IFI16 is a DAMP that activates inflammatory response through TLR4. In addition, if binds to small amounts of LPS through the HIN-B domain enhancing LPS capacity to activate TLR4. The study is original, novel and extremely well organized. Biochemical experiments are extensive and convincing. The paper is well-written and well referenced.*

***Reviewer #3****: In this study, the authors suggest that IFI16 interacts with LPS and TLR4 to promote inflammatory cytokine expression. This idea is interesting and the authors provide some interesting biochemical data to support the interactions proposed. However, the functional significance of these interactions (presented in Figure 3) are of such minor significance that I am not convinced by the conclusions offered. The data presented shows very modest effects of IFI16 on LPS signaling (less than two fold). Whether a 2 fold increase in LPS signaling is of importance in any physiological setting is undefined. For these reasons, I cannot support the conclusions of this study. Much more mechanistic and functional analysis in vitro and in vivo would need to be performed before the model proposed could be considered solid enough for publication. One minor comment is indicated below.*

*1. Line 75 states that LPB transfers LPS to CD14. This statement is incorrect. Elegant studies from Weiss and colleagues demonstrated that LBP binds to LPS and enables CD14 to extract a monomer of this PAMP. LBP does not transfer LPS, it allows CD14 to extract LPS. Please correct this statement and cite the appropriate publications.*

We thank Reviewer #3 for pointing out this inaccuracy. The sentence has been accordingly revised, appropriate citations have been included, and it now reads as (pages 3-4, lines 75-80): “Serum LPS is recognized by the LPS binding protein (LBP), which then forms transient ternary complexes with soluble or membrane-anchored CD14 (sCD14 or mCD14, respectively). Subsequently, CD14 dissociates from LBP to extract monomeric LPS (Gioannini and Weiss, Immunol Res 2007; Ryu et al., Immunity 2017), and through mCD14, LPS is finally presented to the TLR4/MD2 complex, thereby leading to the activation of multiple signaling components, including NF-κB and IRF3, which in turn transcriptionally activate proinflammatory cytokines (Park and Lee, Exp Mol Med 2013).”

**Part II – Major Issues: Key Experiments Required for Acceptanc**e
**Reviewer #1**:

*1. The results are potentially interesting. However, some evidence that these interactions and effects can be observed using IFI16 released from immune cells seems required to ensure that the results are relevant and not just representing an in vitro artifact of overexpressed recombinant agents.*

We fully agree with Reviewer #1 that there might be some structural or functional differences between endogenous and bacterial IFI16, which may eventually result in different biological behaviors. To rule out this possibility and add this crucial control to the manuscript, we set up an *in vitro* experimental model that allowed us to test the biological activity of endogenous IFI16 released by cells under stress stimuli. Briefly, we stimulated THP-1 cells with supernatants from UVB-treated U2OS or IFI16 knockout U2OS (U2OS-IFI16^-/-^) cells and assessed their secretion of pro-inflammatory cytokines.

In good agreement with our previous findings (Costa et al., British J Dermatol 2010), UVB treatment (800 Jm^-2^ for 16h) of U2OS led to massive release of IFI16 in the culture supernatants, but it failed to do so in U2OS-IFI16^-/-^ cells (new Fig 6A), thus providing us with IFI16-containing and -depleted medium, respectively. Subsequently, we incubated THP-1 cells with an aliquot of each conditioned medium and assess after 24 h their production of pro-inflammatory cytokines by ELISA. Consistent with the results obtained with the recombinant IFI16 protein, the conditioned medium from UVB-treated U2OS cells significantly enhanced the release of IL-6, IL-8 and TNF-α by THP-1 cells when compared to mock-treated cells—*i.e.,* 20.9-fold higher for IL-6; 83-fold for IL-8; and 33.4-fold for TNF-α (new Fig 6B). Importantly, cytokine release was significantly lower in THP-1 cells treated with supernatants from UVB-treated U2OS-IFI16^-/-^ cells when compared to the UVB-treated normal counterpart—*i.e*., 2.8-fold lower for IL-6; 2.7-fold for IL-8; and 2.6-fold for TNF-α—, indicating that the effects observed could be ascribed to the IFI16 protein present in the supernatant. To corroborate these data, anti-TLR4 antibodies were added to THP-1 cells for 10 min before exposing them to the conditioned medium. As shown in new Fig 6B, anti-TLR4 treatment of THP-1 cells led to a significant drop in cytokine production.

Altogether, these results seem to rule out the possibility of artifacts in our experimental conditions.

These data have been included in the Results section (pages 11-12, lines 270-280).

*2. Analysis of individual domains of IFI16 supports that the HIN-B domain is important for the recognition of LPS. To verify this, they should show that IFI16 lacking the HIN-B domain does not interact with LPS to promote cytokine production*.

We thank the Reviewer for suggesting this important experiment. To ascertain the role of the HIN-B domain in our system, we generated an IFI16 variant lacking the HINB domain (IFI16ΔHINB) that we used to perform *in vitro* pull-down assays with biotinylated LPS-EB, as already done with full-length IFI16. As shown in new S1B Fig, the IFI16ΔHINB variant failed to co-precipitate in the presence of biotinylated LPS, confirming that the HINB domain is indeed required for IFI16 binding to LPS.

In addition, to further strengthen the biological relevance of the interaction between the IFI16 HINB domain and LPS, we used this variant to stimulate both THP-1 and 786-O cells, alone or pre-complexed with LPS-EB as previously done, and then assess the induction of pro-inflammatory cytokines after 24 h. As shown in new Fig 3A and B, even though the IFI16ΔHINB protein maintained an activity similar to the full-length protein, the addition of LPS-EB did not increase its proinflammatory activity in both 786-O and THP-1 cells. Altogether, these findings clearly demonstrate that the HINB domain is essential for the interaction with LPS.

These data have been included in the Results section (page 8, lines 177-180; page 9, lines 213-217 and 220-223).

3. *Can the authors exclude that DNA has an impact on the outcome of the Co-IPs? It has been reported that both LPS and IFI16 may bind to DNA*.

We thank the Reviewer for these thoughtful comments. To clarify this important point and ensure that residual DNA potentially present in the protein extracts did not cause any bias, we performed all Co-IPs using whole-cell extracts treated or not with DNase. As shown in new Fig 7A, the interaction between IFI16 or IFI16/LPS and TLR4 was maintained also in protein extracts obtained from DNase treated cells, indicating that the interaction between these molecules is not mediated by any binding to DNA. A sentence describing these findings has been included in the manuscript (page 13, lines 301-305).

**Reviewer #2**: *The Biochemical experiment are flawless and very convincing*

**Reviewer #3**: *see above*

**Part III – Minor Issues: Editorial and Data Presentation Modifications**

**Reviewer #1**:

*1. Fig. 2E: lanes should be numbered.*

Numbers have been added in new Fig 2E.

*2. The quantities of IFI16 and LPS used for complex formation are very different (i.e. 25 µg/ml and 25 ng/ml). Stoichiometries should be discussed. In this regards it would also be more informative to provide µM rather than µg/ml.*

As the aim of this work was to demonstrate that subtoxic concentrations of LPS, which are known to be present in various pathological settings other than gram-negative infections, are enough to potentiate the pro-inflammatory action of IFI16, we have deliberately chosen to use high doses of IFI16 protein and low doses of LPS.

With regard to expressing LPS concentrations in µM instead of µg/ml, we would rather not change what we think is a more conventional measurement (Deng et al, Immunity 2018; Di Gioia et al, Nat Immunol 2020; Evavold et al, Immunity 2018; Facchini et al, J Med Chem 2018). Indeed, it is more common to express the LPS concentration in µg/ml because it is quite difficult to gather precise data about the molecular weight of this molecule given its ability to form aggregates of various sizes (Jann et al, Eur J Biochem 1975; Srimal et al, Biochem J 1996; Steimle et al., J Med Microbiol 2016).

*3. More detail should be provided on the conditions and kinetics of complex formation*

We concur with the Reviewer that a more detailed description of the conditions and kinetics of complex formation would further strengthen our manuscript. To this end, we have performed SPR-based analysis (now included as Fig 1D in the revised manuscript) using IFI16 as ligand and LPS-EB as analyte, which allowed us to determine both association (K_a_ = 1.13*10^4^ 1/Ms) and dissociation (K_d_ = 1.94*10^-3^ 1/s) rate constants. Our analysis suggests that IFI16, in our experimental setting, binds to LPS with a 1:1 stoichiometry. The reliability of the kinetic constants calculated by assuming a 1:1 binding model is supported by the quality assessment indicator values (Chi^2^ and U value). Furthermore, the results from the saturation binding experiments show that the interaction between IFI16 and LPS follows a prototypical associative binding, with increasing binding rate up to the plateau phase following addition of increasing amounts of the analytes. These new details are reported in the Results section (page 5, lines 110-113) and in the Discussion section (page 15, lines 366-367).

*4. It seems that Figs. 1E and 5D are not mentioned in the text.*

Thank you very much for pointing this out. These Figures (1E is now 1F) are now properly mentioned in the text (page 6, line 133, and page 11 line 264, respectively).

*5. Fig. 6 A-B, IL-6 panel: stimulation of THP-1 cells with IFI16 with either DPLA or MPLA shows only a modest effect of IL-6 gene expression but clearly increases release of this cytokine. This should be discussed.*

We agree with the Reviewer that IL-6 gene enhancement upon stimulation with IFI16 complexed with either DPLA or MPLA is higher at the protein level when compared to the mRNA level. As this trend is frequently seen in THP-1 cells stimulated with the IFI16 protein alone (see Fig 3 panel A *vs.* B), and given that mRNA levels were measured at 24 h post-treatment concomitantly with the ELISA, it is possible that the mRNA levels might have reached a peak a little earlier, thus explaining the accumulation of the protein in the supernatants. As both values are always reported in the manuscript, we believe that this point should not require further discussion. We hope the Reviewer will concur with us.

*6. Fig. 7 A-B: It should be specified to which significant differences the stars are referring. When IFI16 and LPS-EB were added to the cells without pre-incubation, cytokine induction was lower than that measured with IFI16. Does this mean free LPS inhibits the effect of IFI16?*

The significant differences the Reviewer is referring to are relative to each treatment compared with IFI16-treated cells. The figure legends to these figures have been modified accordingly to make this concept clearer. It now reads as: “The *P* values refer to comparisons with IFI16-treated cells (**P* < 0.05, ***P* < 0.01, ****P* < 0.001; one-way ANOVA followed by Dunnett’s test).” (page 43, lines 1034-1035).

With regard to the question *“Does this mean free LPS inhibits the effect of IFI16*”, we think it may be ascribed to a competition effect for the following reasons: *i)* the binding kinetics revealed by SPR analysis clearly indicate that LPS-EB has a much higher affinity for TLR4 and a very slow kinetics of dissociation when compared to the IFI16 protein alone; *ii)* free LPS can indeed successfully compete with IFI16 for TLR4 binding; and *iii)* the resulting inflammatory response is mediated by free LPS, which is known to be a very weak proinflammatory agent at the concentration used. A sentence has been added in the Discussion to clarify these concepts (page 18, lines 443-445).

**Reviewer #2**:

*1. The authors show that LPS binds the HIN-B domain of IFI16 but do not elaborate on why only this domain can bind. What is the difference between the HIN-A and the HIN-B domains? Is there any amino acid motif that could be responsible for binding LPS? Can this motif be found in any other protein of the same family (ALRs?)*

As outlined in the Discussion (page 16, lines 377-382), different biological behaviors of the HINA *vs.* HINB domain have been already reported in other settings—*e.g.* different binding affinities of the IFI16 HINA and HINB domains for the sugar-phosphate backbone of DNA structures (Unterholzner et al., Nat Immunol 2010). In addition, structural analysis of the HIN domain:DNA complexes revealed that, regardless of the high primary and secondary homologies between HINA and HINB, specific spatial shift and lack of amino acid residues might explain the lower DNA-binding affinities observed for the HINA domain when compared to the HINB moiety (Jin et al., Immunity 2012). Thus, it is not surprising that the two HIN domains can also differentially interact with other PAMPs such as bacterial LPS.

We agree with this Reviewer, that in-depth computational analyses would allow us to gain important insights into the binding motif responsible for HINB-LPS interaction. In this regard, we have found that, unlike the HINA domain, the HINB domain harbors an amino acid region (577-637) carrying some degree of homology with LPS-binding motifs found in two other LPS binding proteins, namely lipopolysaccharide-binding protein (LPB) and bactericidal/permeability increasing protein (BPI) (Denlinger et al., J Immunol 2001). Considering that it would require a lengthy investigation to demonstrate that these motifs are responsible for the binding, and that such analysis will be the objective of an already planned research project, we would rather not disclose these computational speculations in this manuscript. We hope the Reviewer will understand our position.

*2. Is there any polymorphism in this motif that is associated to inflammatory or infectious disease in the population? The authors should expand their speculations in the discussion and propose scenarios in which disruption of this interaction may relate to human diseases*

A series of IFI16 polymorphisms have been actually reported and associated—without providing any mechanistic explanation—with inflammatory or infectious diseases (Booiman & Kootstra, Int J Immunogenetics 2014; Marchesan et al, J Periodontol 2017; Eriksson et al., J Immunol 2017). In most cases, they were found in the 5’ or 3’ untranslated regions of the IFI16 gene. However, as we do not know exactly the peptide motif responsible for LPS binding and its potential polymorphism in humans, we are not in a position to make any speculations at this stage. In addition, our working hypothesis is that the pivotal IFI16-based pathogenetic mechanism is due to its aberrant expression and release in the extracellular milieu under inflammatory conditions rather than caused by structural modifications.

**Reviewer #3:** (No response)
