# Supplementary material for: Toll-like receptor 4-mediated inflammation triggered by extracellular IFI16 is enhanced by lipopolysaccharide binding
Source: PLoS Pathog. 2020 Sep 9;16(9):e1008811. doi: 10.1371/journal.ppat.1008811 (PMC7505474; doi:10.1371/journal.ppat.1008811)
Supplement: Attachment — Submitted filename: Iannucci et al_Response to Reviewers_rev2.docx [file ppat.1008811.s006.docx]

***1. Page 11 states that IFNb was not detected when cells were stimulated with LPS but this is contrary to what's known about LPS/TLR4 signaling. Please provide an explanation for this anomaly.***

As stated in the manuscript (page 11, lines 254-256), upon LPS stimulation, TLR4 induces two independent signaling pathways regulated by either the TIRAP/MyD88 or TRAM/TRIF pair of adaptors, which promote the production of proinflammatory cytokines and type I interferons (IFN-I), respectively (Kagan, Trends Immunol. 2017; Takeda and Akira, Semin. Immunol. 2017). The former promotes the nuclear factor-kB (NF-kB)- and activator protein 1 (AP-1)-dependent inflammatory responses that typify TLR4 signal transduction, while TRIF-mediated signaling occurs from endosomes and involves ligand dependent movement of the TLR4 complex from the plasma membrane to the endosomes. This process of receptor endocytosis is critical for regulating TLR4-mediated type I IFN production and is very much dependent on the cell-type intrinsic capacity to internalize TLR4 and induce the expression of TRIF-dependent genes (Brubaker et al., Annu. Rev. Immunol 2015). As TLR4 endocytosis is CD14-mediated (Zanoni et al., Cell 2011), it is well known that undifferentiated THP-1 cells -that express low CD14 levels- produce very low amount of IFN when compared to differentiated THP-1 cells (Petes et al., J Biol Chem 2018). In our study, only undifferentiated THP-1 cells have been used. With regards to 786-O cells, their response to LPS is not so well characterized, but it is not unexpected that one adaptor set for TLR signaling, TIRAP/MyD88 in this case, turned out to be more active/relevant than the other in our setting.

References

Brubaker SW, Bonham KS, Zanoni I, Kagan JC. Innate Immune Pattern Recognition: A Cell Biological Perspective. Annu Rev Immunol. 2015;33: 257–290. doi:10.1146/annurev-immunol-032414-112240

Kagan JC. Lipopolysaccharide Detection across the Kingdoms of Life. Trends Immunol. 2017;38: 696–704. doi:10.1016/j.it.2017.05.001

Petes C, Mintsopoulos V, Finnen RL, Banfield BW, Gee K. The effects of CD14 and IL-27 on induction of endotoxin tolerance in human monocytes and macrophages. J Biol Chem. 2018;293: 17631–17645. doi:10.1074/jbc.RA118.003501

Takeda K, Akira S. TLR signaling pathways. Semin Immunol. 2004;16: 3–9.

Zanoni I, Ostuni R, Marek LR, Barresi S, Barbalat R, Barton GM, et al. CD14 controls the LPS-induced endocytosis of Toll-like Receptor 4. Cell. 2011;147: 868–880. doi:10.1016/j.cell.2011.09.051

***2. Figure 5A x-axis labels read "siTRL4". Please correct.***

Thank you very much for pointing out that. The labels have been modified.
